# Investigating the Role of Free-living Amoebae as a Reservoir for *Mycobacterium ulcerans*

**DOI:** 10.1371/journal.pntd.0003148

**Published:** 2014-09-04

**Authors:** Nana Ama Amissah, Sophie Gryseels, Nicholas J. Tobias, Bahram Ravadgar, Mitsuko Suzuki, Koen Vandelannoote, Lies Durnez, Herwig Leirs, Timothy P. Stinear, Françoise Portaels, Anthony Ablordey, Miriam Eddyani

**Affiliations:** 1 Bacteriology Department, Noguchi Memorial Institute for Medical Research, Accra, Ghana; 2 Evolutionary Ecology Group, Department of Biology, University of Antwerp, Antwerp, Belgium; 3 Department of Microbiology, University of Melbourne, Melbourne, Victoria, Australia; 4 Department of Microbiology, Monash University, Victoria, Australia; 5 Parasitology Department, Noguchi Memorial Institute for Medical Research, Accra, Ghana; 6 Department of Biomedical Sciences, Institute of Tropical Medicine, Antwerp, Belgium; University of Tennessee, United States of America

## Abstract

**Background:**

The reservoir and mode of transmission of *Mycobacterium ulcerans*, the causative agent of Buruli ulcer, still remain a mystery. It has been suggested that *M. ulcerans* persists with difficulty as a free-living organism due to its natural fragility and inability to withstand exposure to direct sunlight, and thus probably persists within a protective host environment.

**Methodology/Principal Findings:**

We investigated the role of free-living amoebae as a reservoir of *M. ulcerans* by screening the bacterium in free-living amoebae (FLA) cultures isolated from environmental specimens using real-time PCR. We also followed the survival of *M. ulcerans* expressing green fluorescence protein (GFP) in *Acanthameoba castellanii* by flow cytometry and observed the infected cells using confocal and transmission electron microscopy for four weeks *in vitro.*

IS*2404* was detected by quantitative PCR in 4.64% of FLA cultures isolated from water, biofilms, detritus and aerosols. While we could not isolate *M. ulcerans*, 23 other species of mycobacteria were cultivated from inside FLA and/or other phagocytic microorganisms. Laboratory experiments with GFP-expressing *M. ulcerans* in *A. castellani* trophozoites for 28 days indicated the bacteria did not replicate inside amoebae, but they could remain viable at low levels in cysts. Transmission electron microscopy of infected *A. castellani* confirmed the presence of bacteria within both trophozoite vacuoles and cysts. There was no correlation of BU notification rate with detection of the IS*2404* in FLA (r = 0.07, n = 539, p = 0.127).

**Conclusion/Significance:**

This study shows that FLA in the environment are positive for the *M. ulcerans* insertion sequence IS*2404.* However, the detection frequency and signal strength of IS*2404* positive amoabae was low and no link with the occurrence of BU was observed. We conclude that FLA may host *M. ulcerans* at low levels in the environment without being directly involved in the transmission to humans.

## Author Summary

*Mycobacterium ulcerans*, the causative agent of Buruli ulcer (BU) is an environmental pathogen known to reside in aquatic habitat. However, the reservoir and modes of transmission to humans still remain unknown. *M. ulcerans* can probably not live freely due to its natural fragility and inability to withstand exposure to direct sunlight. This study investigated the hypothesis that free-living amoebae (FLA) can serve as a reservoir of *M. ulcerans* by testing for its presence in amoebae isolated from water bodies in BU endemic and non-endemic communities and whether the pathogen can remain viable when experimentally infected in amoebae in the laboratory. We detected only one (IS*2404*) of the three (IS*2606* and KRB) targets for the presence of *M. ulcerans* in amoebae cultures and found no correlation between its presence in the environment and BU notification rate. *M. ulcerans* remained viable at low levels in amoebae for 28 days *in vitro*. We therefore conclude that FLA may host *M. ulcerans* at low levels in the environment without being directly involved in the transmission to humans.

## Introduction

*Mycobacterium ulcerans* is a slow growing environmental pathogen responsible for a necrotizing cutaneous infection called Buruli ulcer (BU). The disease has been reported in over 30 countries worldwide mainly in tropical and subtropical climates and emerged as an increasing cause of morbidity in endemic rural communities in some West and Central African countries with Benin, Côte d'Ivoire and Ghana bearing the highest burden of disease [1].

Most BU endemic areas are found close to slow flowing or stagnant water bodies and it is therefore assumed that the aquatic ecosystem may be a source of *M. ulcerans* from which the bacterium is transmitted to humans. This is supported by several studies that have detected *M. ulcerans* DNA sequences in a variety of environmental specimens including fish, snails, detritus, biofilms, soil, water filtrands, insects and protozoa [2]–[5]. Recently in Australia, *M. ulcerans* DNA has been detected in mosquitoes, faecal matter and skin lesions of small terrestrial mammals (ringtail and brushtail possums) that are thought to harbor and vector the bacterium [6], [7]. However, the main reservoir and modes of transmission of BU outside Australia still remain unknown.

Since the discovery that *Legionella pneumophila* is able to infect and replicate in free-living amoebae (FLA) [8], there has been an increasing number of studies on the role of FLA in the survival of pathogenic organisms [9]. Also, several species of mycobacteria (*M. shottsii*, *M. pseudoshottsii*, *M. tuberculosis*, *M. leprae*, *M. ulcerans*, *M. marinum*, *M. bovis*, *M. avium subsp paratuberculosis* and *M. avium*) have been shown to survive within protozoa [4], [10]–[16]. *M. ulcerans* bears characteristic genomic signatures that are typical of host restricted pathogens suggesting that *M. ulcerans* is unlikely to be free-living in the environment but is instead undergoing or has undergone adaptation to a specific ecological niche [17]. Internalization of infectious agents inside other parasites is a recurring theme in biology and represents an evolutionary strategy for survival that may sometimes enhance pathogenesis or transmissibility [18]: Bacteria “hidden” in their protozoan hosts may more easily infect vertebrate end hosts, multiplying within protozoans to escape immune reactions [15], [18].

Water bodies in areas of high BU endemicity have been reported to contain significantly more FLA than in low endemic areas [19]. Recently, we demonstrated that *M. ulcerans* can be phagocytosed *in vitro* by *Acanthamoeba polyphaga* and persist for at least 2 weeks [4]. This study also showed a higher detection frequency of the IS*2404* target in FLA cultures as compared to crude samples from the environment. The aim of the present study was to further explore FLA as a reservoir for *M. ulcerans* by screening *M. ulcerans* in FLA from aquatic environment sampled for 10 months and relating this to the BU notification rate in the same endemic area. Furthermore, we experimentally investigated the ability of *M. ulcerans* to survive and replicate within *A. castellanii* by infecting these amoebae with *M. ulcerans* expressing green fluorescence protein (GFP).

## Materials and Methods

### Study sites and specimen collection

**Figure 1:**
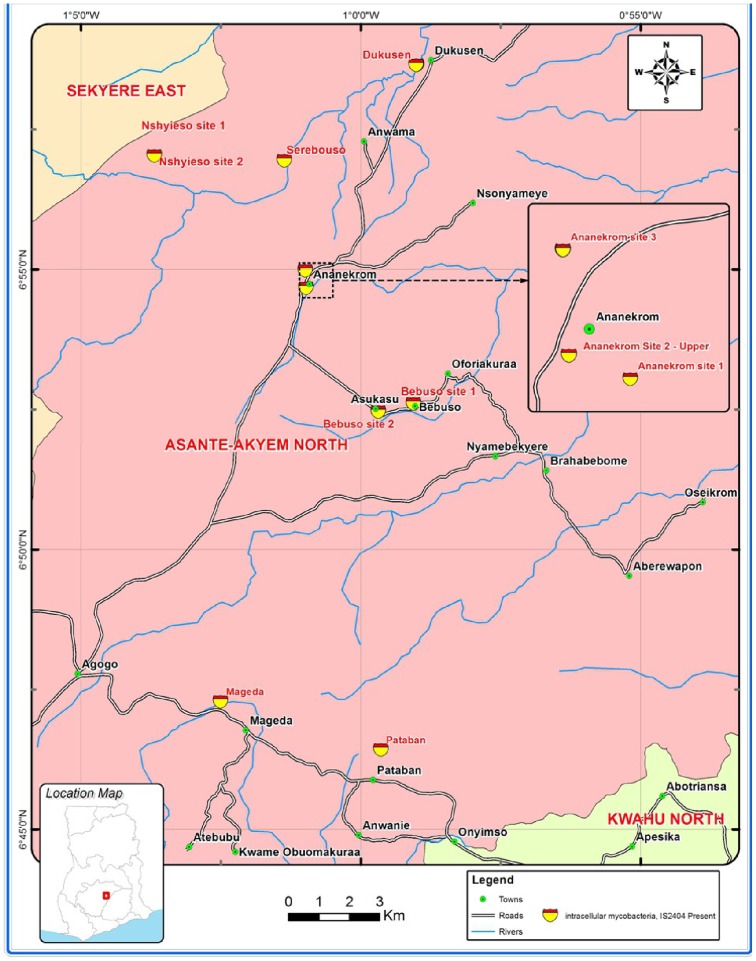
Distribution of the IS*2404* target and intracellular mycobacteria from the sampling sites.

The study was carried out in five endemic communities (with recorded human BU cases): Ananekrom, Nshyieso, Serebouso, Dukusen and Bebuso, and two non-endemic communities (no recorded human BU cases): Mageda and Pataban in the Asante Akim North Municipal of Ghana (Table 1, Fig. 1). These communities are on average 18 km apart and were selected based on number of BU cases reported at the Agogo Presbyterian Hospital (APH), the Municipal health facility serving all communities (Fig. 1). Week-long monthly field visits were made for 10 months between October 2008 and July 2009 to randomly collect environmental specimens: water, biofilm from plants and tree trunks, detritus and aerosols. The specimens were taken between 6:00 am and 8:00 am, the peak period of human contact activities in the water bodies. Biofilms (n = 428) were taken by scraping surfaces of tree trunks, floating logs and tree stumps with sterile scalpels and cotton swabs into 50 mL sterile Falcon tubes. Detritus (n = 45) were scooped by hand into 50 mL sterile Falcon tubes. Water specimens (n = 53) were taken from mid column with buckets of which 3–10 liters were concentrated via 0.45 µm membrane filters (Sartorius Stedim Biotech GmbH, Germany) depending on the turbidity of the water. During the last two months of sampling, non-nutrient agar (NNA) plates (n = 13) seeded with *Escherichia coli* were exposed for 30 minutes for isolation of FLA from aerosols generated next to the water bodies.

**Table 1:** Information on sampling sites.

				Clinically diagnosed human cases positive by IS*2404* PCR			
Communities	Water body	Activities	Population size	2008 (%)	2009 (%)	2010 (%)	2011 (%)
Ananekrom	Egyaah River upper and lower, Betom River	Fishing, recreation, pathway for humans and animals, *domestic activities	1951	28 (1.44)	31 (1.59)	39 (2.00)	33 (1.69)
Nshyieso	Esuo-Efi River (stagnant water)	*Domestic activities	1429	8 (0.56)	10 (0.70)	5 (0.35)	17 (1.19)
Serebouso	Onwamtifi River	*Domestic activities	1275	7 (0.55)	16 (1.25)	11 (0.86)	6 (0.47)
Dukusen	Onwam River	*Domestic activities	675	4 (0.59)	2 (0.30)	7 (1.04)	2 (0.30)
Bebuso	Pupunasoe River (stagnant water)	*Domestic activities, human and animal crossing, fishing	966	6 (0.62)	4 (0.41)	5 (0.52)	4 (0.41)
Mageda	Abena Supuni River	Market traders drink from it	773	0 (0)	0 (0)	0 (0)	0 (0)
Pataban	Pataban River	Drinking	1421	0 (0)	0 (0)	0 (0)	0 (0)

*Domestic activities: bathing, washing, cooking and sometimes drinking.

### Specimen processing

Seven milliliters phosphate buffered saline (PBS) were added to the membrane filters (cut into smaller pieces), swabs and scalpels contained in 50 mL Falcon tubes and shaken vigorously to dislodge the substrate and biofilms from the surface. For detritus, specimens were processed as described by Gryseels *et al.* [4].

### Isolation of FLA

A piece of the membrane filter and 2 to 3 drops of the suspensions (biofilms and detritus) were inoculated at the centre of 1.5% NNA plates seeded with *E. coli* for cultivation of FLA [20] at 28.5°C. The inoculated NNA plates were examined daily for the presence of trophozoites and cysts using the 10× objective of a bright field microscope. When FLA were observed, they were subcultured on new NNA plates seeded with *E. coli* [21]. After 3 or 4 subcultures, the FLA were harvested by scraping the surface with an inoculating loop and suspending them in 1.5 mL sterile distilled water.

### DNA preparation

The modified Boom method was used for the extraction of DNA from FLA cultures as described previously [22], [23].

### Detection of *M. ulcerans* DNA

Two multiplex real-time PCR assays were performed on the DNA extracts to test the presence of three distinct sequences: IS*2404*, IS*2606* and the ketoreductase B (KRB) domain in the *M. ulcerans* genome as described by Fyfe *et al.* [24]. All DNA extracts were first screened for the IS*2404* target multiplexed with an internal positive control to check for PCR inhibitors such as humic and fulvic acids (commonly found in environmental specimens) [24]. The second PCR assay for detecting the presence of IS*2606* and KRB was done on FLA cultures that turned out positive for the IS*2404* target. Amplification and detection was carried out using the 7500 real-time PCR system (Applied Biosystems).

### Identification of FLA

The identification of FLA of the genera *Acanthamoeba*, *Naegleria* and *Vahlkampfiidae* was confirmed using the primer sets JDP1/JDP2, ITSfw/ITSrv and JITSfw/JITSrv respectively as described by Gryseels *et al.* [4] and Eddyani *et al.* [19].

### Isolation of *M. ulcerans* and other intracellular mycobacteria from specimens

Specimens were processed to kill extracellular mycobacteria as described by Gryseels *et al.* [4], decontaminated using the oxalic acid method [25], inoculated on LJ medium, and incubated at 30°C with weekly examination for a year. Supernatants containing extracellular mycobacteria were cultured for week at the same temperature to monitor the effectiveness of the isolation method.

### Identification of intracellular mycobacteria

Direct smear examination (DSE) by ZN staining for acid fast bacilli (AFB) was performed on single colonies grown on LJ media. Cultures positive for AFB were subjected to a nested PCR specific for the mycobacterial 16S rRNA gene [26], [27] after heat-inactivation of mycobacterial suspensions in TE for 10 minutes. Amplicons were sequenced by the VIB Genetic Service Facility (Antwerp, Belgium). The sequenced data were compared with known sequences in the GenBank database and interpreted using the BlastN algorithm (available on http://www.ncbi.nlm.nih.gov/BLAST/). The 16S rRNA sequences were also matched against entries in the RIDOM (Ribosomal Differentiation of Medical Microorganisms) database (http://www.ridom-rdna.de/). Direct detection of mycobacterial DNA in FLA cultures was done using the same 16S rRNA PCR assay.

### Infection of *A. castellanii* with *M. ulcerans*

*M. ulcerans* strains JKD8083 (which expresses GFP) and 04126204 (which does not express GFP) were grown in 7H9 broth or 7H10 agar supplemented with OADC (Difco). Real-time PCR was performed on *M. ulcerans* strains to estimate cell numbers as previously described [28]. Primers targeting the 16S rRNA gene were used for detection of mycobacteria. Known amounts of *M. ulcerans* Agy99 genomic DNA were used to construct a standard curve and cell numbers were estimated based on the predicted mass of an *M. ulcerans* chromosome [24].

*A. castellanii* was cultured in peptone-yeast extract-glucose (PYG) medium at 22°C in the dark as described by Moffat and Tompkins [29]. Trophozoites were harvested at 400× *g* for 10 minutes (Eppendorf, 5810R) and adjusted to a final concentration of 10^6^ cells ml^−1^ in *Acanthamoebae* (AC) buffer as described [29].

Bacterial strains were concentrated by centrifugation at 6000× *g* for 15 minutes at room temperature and then resuspended in AC buffer. A preparation of 1×10^6^ cells of *A. castellanii* was mixed with 1×10^6^ cells of either *M. ulcerans* (JKD8083) or (04126204) in 20 mL PYG broth and incubated at 22°C for 30 minutes. Co-cultures were washed three times in AC buffer and treated with amikacin (150 µg/ml) as described [30] to kill extracellular bacteria. Trophozoites were then washed and resuspended in 50 mL AC buffer. Three milliliters samples were taken at 1, 2, 7, 14, 21 and 28 days for analysis.

### Flow cytometry

At each time point, samples were washed with FACS buffer six times before a final resuspension in 500 µl of FACS buffer. FACS was carried out using uninfected amoebae to identify trophozoite populations. The subsequent infected samples were gated only on these relevant populations. All samples, including the controls (uninfected amoebae and bacteria only) were analyzed with a FACS (Becton Dickinson) equipped with a 488 nm argon laser. At each time point 50,000 events were counted. Background fluorescence was determined by using infections of *A. castellanii* with non-fluorescent *M. ulcerans* (04126204). Percentages of fluorescing amoebae were then calculated using Flowjo (v8.7).

### Confocal and electron microscopy

Aliquots of infected amoebae in AC buffer were again pelleted and washed in 1× PBS before DAPI staining according to the manufacturer's instructions (Invitrogen). Samples were imaged using a LAS700 confocal microscope (Zeiss) with a 100× oil immersion lens.

At different time points 1 mL aliquots were used to examine for *M. ulcerans* within *A. castelanii* as described previously [31] using the electron microscope.

### Statistical analysis

Statistical analyses were performed in SPSS 18.0 (SPSS Inc., Chicago, IL) software. Standard multiple regression was used to investigate whether the isolation frequency of FLA in communities (water bodies) was related to the waterbody-specific prevalence of the IS*2404* target and mycobacterial DNA (in those FLA). Hierarchical multiple regression was also used to assess the ability of some parameters (detection of IS*2404* positive FLA, detection of mycobacterial DNA in FLA, and isolation of intracellular mycobacteria) to predict BU notifications (number of reported cases/number of inhabitants/month), after controlling for the influence of time. Logistic regression was performed to assess the influence of time (months) on the detection of IS*2404* in FLA, isolation of intracellular mycobacteria and frequency of isolated FLA. The relationship between the isolation of FLA and the type of specimen and the communities sampled was investigated using the Pearson product-moment and Spearman Rank Order correlation (rho) coefficient. Kruskal-Wallis and Mann-Whitney U Tests were used to compare the isolation frequency of FLA and detection frequency of IS*2404* between the different types of specimen and communities. P values <0.05 were considered significant.

## Results

### Isolation and identification of FLA

Five hundred and thirty nine environmental specimens were collected from October 2008 to July 2009. FLA were cultured from 405 (75.10%) specimens. Confirmation using three different PCR primer sets permitted the classification into three genera of FLA from 370 (68.65%) specimens with some cultures harboring more than one genus of FLA (*Acanthamoeba* [n = 157], *Vahlkamfiidae* [n = 306] and *Naegleria* [n = 118]) (Table S2). FLA were isolated from all specimen types (Table 2), and showed a statistically significant difference across the specimen types, (χ^2^ (4, n = 539) = 14.532, p = 0.006) with aerosols recording the highest mean rank (354.50) compared to the other specimen types. A Mann-Whitney U Test also showed that FLA were more frequently isolated from aerosols than plant biofilm (p = 0.006) (Bonferroni adjustment alpha level = 0.008). FLA were isolated from all communities, the isolation frequency showed a significant difference across communities ((χ^2^ (6, n = 539) = 14.955, p = 0.021) with Nshyieso recording the highest mean rank (308.22). A Mann-Whitney U Test showed no significant difference in FLA isolation between Mageda and Nshyieso (p = 0.878) but showed a difference between Nshyieso and the communities Dukusen, Ananekrom and Serebouso (p = 0.000, p = 0.002, p = 0.006).

**Table 2:** Isolation of FLA per type of specimen and sampling site.

Sampling sites	Aerosols (%)	Biofilm (%)	Detritus (%)	Water (%)	Total (%)
Ananekrom site 1	1/1 (100)	41/70 (58.57)	4/4 (100)	9/11 (81.82)	55/86 (63.95)
Ananekrom site 2-upper	0/0 (0)	1/2 (50.0)	1/1 (100)	0/0 (0)	2/3 (66.67)
Ananekrom site 2-lower	0/0 (0)	0/2 (0)	1/1 (100)	0/0 (0)	1/3 (33.33)
Ananekrom site 3	0/0 (0)	3/4 (75)	2/2 (100)	0/1 (0)	5/7 (71.43)
Bebuso site 1	1/1 (100)	33/54 (61.11)	2/3 (66.67)	5/7 (71.43)	41/65 (63.08)
Bebuso site 2	2/2 (100)	19/24 (79.17)	0/0 (0)	2/2 (100)	23/28 (82.14)
Dukusen	4/4 (100)	52/88 (59.09)	6/8 (75)	5/10 (50.0)	67/110 (60.91)
Mageda	0/0 (0)	8/10 (80.0)	5/5 (100)	0/1 (0)	13/16 (81.25)
Nshyieso site 1	0/0 (0)	2/3 (66.67)	2/2 (100)	0/1 (0)	4/6 (66.67)
Nshyieso site 2	1/1 (100)	64/77 (83.12)	4/6 (66.67)	9/9 (100)	78/93 (83.87)
Pataban	0/0 (0)	7/10 (70.0)	4/5 (80.0)	0/1 (0)	11/16 (68.75)
Serebouso	4/4 (100)	51/84 (60.71)	6/8 (75.0)	9/10 (90.0)	70/106 (66.04)
Total	13/13 (100)	281/428 (65.65)	37/45 (82.22)	39/53 (75.58)	370/539 (68.65)

### Detection of *M. ulcerans* DNA in FLA cultures

Twenty five out of 370 FLA cultures obtained from 539 specimens (4.64%) tested positive for the IS*2404* target (Table S1). C_T_ values ranged from 29.46 to 38.05 corresponding to ≤1–10 genomes µl^−1^ DNA extract of *M. ulcerans*. All IS*2404* positive FLA cultures tested negative for the IS*2606* and KRB targets. The IS*2404* target was detected significantly more often in *Acanthamoeba* and *Vahlkampfiidae* (χ^2^ (1, n = 539) = 5.532 p = 0.019, phi = 0.111 and χ^2^ (1, n = 539) = 4.814 p = 0.028, phi = 0.103) than in *Naegleria* (χ^2^ (1, n = 539) = 0.259, p = 0.611, phi = 0.033). None of the mycobacterial cultures isolated intracellularly from six of these specimens tested positive for IS*2404*. IS*2404* positive FLA were detected in all endemic and non-endemic communities (Table 3, Fig. 1). There was no significant difference in detection of the IS*2404* target from FLA across the specimen types (χ^2^ (4, n = 539) = 6.715, p = 0.152).

**Table 3:** Detection of IS*2404* target in FLA cultures per type of specimen and sampling site.

Sampling site	Type of specimen					Total
	Aerosols	Biofilm plant	Biofilm trunk	Detritus	Water	
Ananekrom site 1	0/1 (0)	2/38 (5.26)	0/32 (0)	0/4 (0)	1/11 (9.09)	3/86 (3.49)
Ananekrom site 2-upper		1/1 (100)	0/1 (0)	0/1 (0)		1/3 (33.33)
Ananekrom site 2-lower		0/1 (0)	0/1 (0)	0/1 (0)		0/3 (0)
Ananekrom site 3		0/2 (0)	0/2 (0)	1/2 (50)	0/1 (0)	1/7 (14.29)
Bebuso site 1	0/1 (0)	1/26 (3.85)	2/28 (7.14)	0/3 (0)	0/7 (0)	3/65 (4.62)
Bebuso site 2	0/2 (0)	1/15 (6.67)	0/9 (0)		1/2 (50)	2/28 (7.14)
Dukusen	0/4 (0)	0/49 (0)	1/39 (2.56)	1/8 (12.5)	0/10 (0)	2/110 (1.82)
Mageda		0/5 (0)	0/5 (0)	1/5 (20)	0/1 (0)	1/16 (6.25)
Nshyieso site 1		0/3 (0)		0/2 (0)	0/1 (0)	0/6 (0)
Nshyieso site 2	0/1 (0)	3/38 (7.89)	3/39 (7.69)	0/6 (0)	1/9 (11.11)	7/93 (7.53)
Pataban		0/5 (0)	0/5 (0)	1/5 (20)	0/1 (0)	1/16 (6.25)
Serebouso	2/4 (50)	2/40 (5)	0/44 (0)	0/8 (0)	0/10 (0)	4/106 (3.77)
total	2/13 (15.38)	10/223 (4.48)	6/205 (2.93)	4/45 (8.89)	3/53 (5.66)	25/539 (4.64)

Some of the IS*2404* positive cultures harbored more than one genus of FLA (*Acanthamoeba* [n = 13 (2.4%)], *Vahlkampfiidae* [n = 20 (3.7%)] and *Naegleria* [n = 7 (1.3%)]). The amoebae had similar ITS sequences to those of *Acanthamoeba* sp., *A. lenticulata*, *A. castellanii*, *Naegleria sp.* strain WTP29, *N. lovaniensis*, *N. philippinensis* and *V. avara*, *V. inornata*, *Acanthamoeba* sp. T11 genotype and *Acanthamoeba* spp. T4 genotype as reported by Gryseels *et al.* [4]. Three of the positive cultures could not be identified with the primers used (Table S1).

### Identification of intracellular mycobacteria

We could not cultivate *M. ulcerans* from the FLA, however, other mycobacteria were cultured from intracellular origins in 162 (30.06%) specimens; 109 (67.28%) among these originated from specimens from which we also isolated FLA. All isolates were confirmed by DSE for AFB and partial sequencing of the 16S rRNA gene. There was no growth of bacteria after the supernatants containing the killed extracellular mycobacteria were cultured for a week. One hundred and thirty one isolates showed >99% sequence similarity match with the available database in GenBank (NCBI and RIDOM 16S rDNA) comprising a total of 23 mycobacterial species (Table S3). Eight of the remaining sequence data were too short to be identified and 23 (14.20%) isolates had mixed growth, which made identification impossible. The most frequently isolated species were *M. arupense* (39.69%), *M. fortuitum* (7.63%) and *M. lentiflavum* (4.58%).

After screening the FLA cultures for the mycobacterial 16S rRNA gene, 159 (42.97%) of the 370 were positive but mycobacteria were not identified to the species level.

### Evolution of study parameters during the sampling period

**Figure 2:**
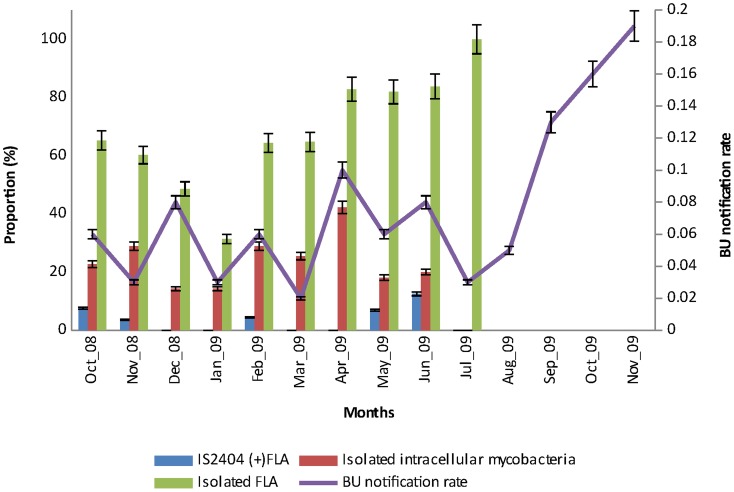
Proportions of FLA isolated from specimens, IS*2404* detected in FLA cultures, mycobacteria isolated from an intracellular source over the sampling period (October 2008–July 2009) and BU notification rate until four months after the sampling period. Error bars on the left Y-axis represent 95% confidence interval (CI) of the proportions of isolated FLA (68.3±1.66), IS*2404* detected in FLA cultures (3.5±0.37) and intracellular mycobacteria (21.49±0.95) sampled monthly from environmental specimens (n = 539) for the specified period. Error bars on the right Y-axis represent the 95% CI of BU notification rate of the 5 endemic communities (0.08±0.04) with a population size of 6,296.

The detection of intracellular mycobacteria peaked in April 2009 followed by a peak in the detection of IS*2404* positive FLA in June 2009 and the isolation of FLA in July 2009. The highest number of BU cases was however reported four months later in November 2009 after FLA isolation peaked (Figure 2).

Time accounted for a 13.80% variance in the BU notification rate (new BU cases per month) using a hierarchical multiple regression model (F (4, 534) = 26.00, p<0.001). The three other parameters, intracellular mycobacteria, detection of IS*2404* target and detection of mycobacterial DNA in FLA, together explained an additional 2.5% of the variance in BU notification rate, after controlling for time, *R* squared change = 0.025, *F* change (3, 534) = 5.251, p<0.001. In the final model, only two parameters were statistically significant, with time recording a higher beta value (beta = 0.312, p<0.001) than detection of mycobacterial DNA in FLA (beta = 0.152, p<0.001). The isolation frequency of FLA varied significantly through time ((χ^2^ (1, N = 539) = 28.479, p<0.001) as well. There was a positive correlation of BU notification rate with detection of mycobacterial DNA in FLA (r = 0.27, n = 539, p<0.0005) but not with detection of the IS*2404* target in FLA (r = 0.07, n = 539, p = 0.127).

Using a direct logistic regression model, time accounted for between 5.1% (Cox and Snell R square) and 7.2% (Nagelkerke R squared) of the variance in FLA isolation but could not predict the variances in the detection of IS*2404* in FLA ((χ^2^ (1, N = 539) = 2.034, p = 0.154) and isolation of intracellular mycobacteria ((χ^2^ (1, N = 539) = 0.132, p = 0.717).

### *M. ulcerans* persists in amoebae for up to 28 days

**Figure 3:**
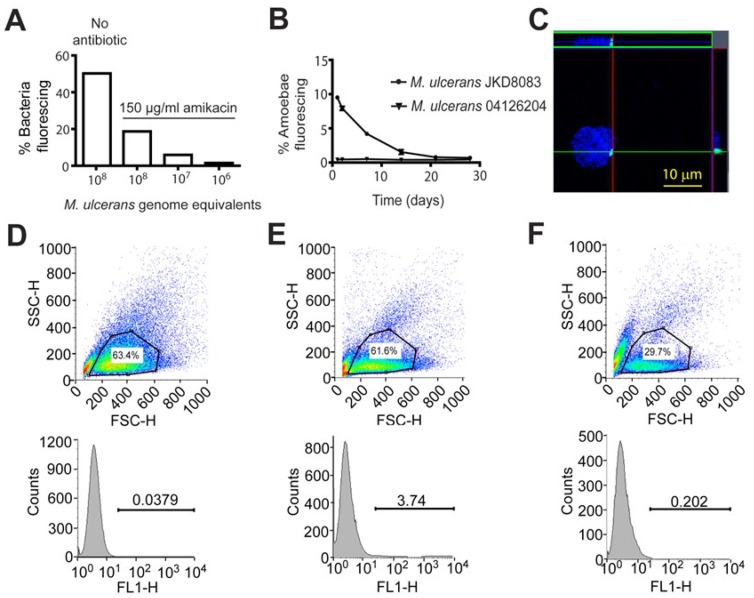
(A) Percentage of fluorescing bacteria present following growth at room temperature for 7 days in AC buffer supplemented with 150 µg/ml amikacin. (**B**) Summary of flow cytometry data of *A. castelanii* infected with fluorescing (JKD8083) and non-fluorescing (04126204) *M. ulcerans* for 28 days. The mean percentage and SD of 3 biological repeats of fluorescent trophozoites at a MOI of 1 are shown. (**C**) Confocal microscopy demonstrating the intracellular location of *M. ulcerans* 3 hours post-infection in a DAPI stained trophozoite. Scale bar indicates 10 µm. (**D–F**) Representative FACS plots indicating forward scatter (x-axis) and side scatter (y-axis) following 7 days of co-incubation of amoebae alone (**D**), *M. ulcerans* JKD8083 (**E**) and *M. ulcerans* 04126204 (**F**). Lower panels show the gated trophozoites and FL1 fluorescence vs counts. Numbers refer to percentage of gated trophozoites fluorescing.

*M. ulcerans* infections of *A. castellanii* were performed over a four week period and quantified using flow cytometry. Methods involving the removal of extracellular bacteria using amikacin have been reported previously [4], [30] and were independently tested here (Figure 3A). *M. ulcerans* JKD8083 bacteria alone (10^6^–10^8^) were treated with amikacin for 7 days to test the effect of the antibiotic on extracellular bacteria. Treatment with 150 µg/ml amikacin for 7 days left 18.7% of 10^8^ extracellular bacteria (5.92% of 10^7^, 1.53% of 10^6^) fluorescing above background (Figure 3A).

Using flow cytometry, 50,000 events were counted at each time point. Amoebae were experimentally infected with *M. ulcerans* by placing them in co-culture at a multiplicity of infection of 1 for 30 min, and killing remaining extracellular mycobacteria with amikacin. At day 0, 9.51% of the amoebae were infected with *M. ulcerans*, but this percentage gradually decreased until at day 28 (end of experiment) only 0.7% of amoebae were infected with *M. ulcerans* (Figure 3B). Figure 3 (D–F) shows gated trophozoites following 7 days co-incubation of amoebae alone (D), *M. ulcerans* JKD8083 (E) and *M. ulcerans* 04126204 (F). Amoebae infected with non-fluorescing bacteria however, fluoresced at levels less than 0.05% over the 28-day time course (Figure 3B), therefore we neglected this background fluorescence.

### An intracellular location of *M. ulcerans* within *A. castellanii*

**Figure 4:**
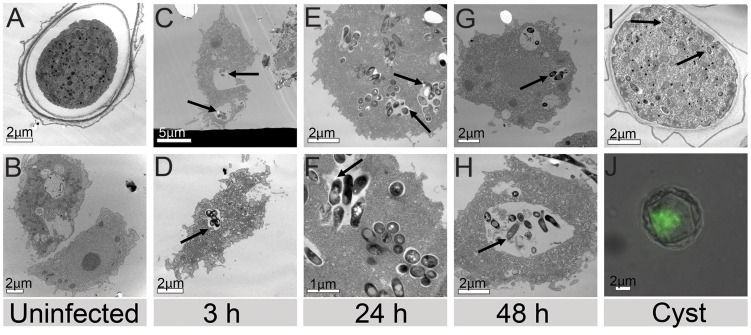
Transmission electron microscopy showing uninfected (A) cysts and (B) trophozoites as well as the intravacuolar location of *M. ulcerans* (arrows) within infected, intact trophozoites (C, D) 3 h, (E, F) 24 h, (G, H) 48 h and (I) cysts 3 weeks post infection. (**J**) is a composite image showing a cyst 22 days post infection by fluorescence microscopy.

Localisation of the bacteria with respect to the amoebae was determined by both fluorescent confocal microscopy and electron microscopy (Figure 3C; Figure 4). Confocal optical sections demonstrate a mycobacterium within *A. castellanii* three hours post infection (Figure 3C). Video S1. shows fluorescing bacteria in *A. castelanii* at 24 h post infection. Examination of 1 mL aliquots of *M. ulcerans*-infected trophozoites and cysts by transmission electron microscopy demonstrated an intravacuolar location for *M. ulcerans* at 3, 24 and 48 hours post infection (Figure 4, Panels C–I). Subsequent microscopy on an extended time series reveals the presence of intracellular bacteria within cysts at 22 days post infection (Figure 4I–J).

## Discussion

It has been suggested that *M. ulcerans* persists with difficulty in the environment as a free-living organism due to its natural fragility [32] and may be maintained in a commensal or parasitic relationship with hosts that protect the bacilli against potentially unfavorable environments. This hypothesis is supported by the observation that *M. ulcerans*, unlike its close environmental relatives, has degraded genome, with many pseudogenes, such as a mutation in the *crt* locus. This locus harbors genes responsible for the production of light-inducible carotenoids that affects its ability to withstand exposure to direct sunlight and hence diminishes its capacity to live freely [17].

A previous study by our group detected the IS*2404* target twice as often in FLA cultures as in environmental specimens [4]. Extending this study, we investigated the role of FLA and other phagocytic microorganisms as a reservoir of *M. ulcerans* in the aquatic environment for 10 months and tested the survival of *M. ulcerans* within *A. castellanii in vitro*.

*Acanthamoeba* sp. and *Vahlkampfiidae* sp. were isolated more frequently than *Naegleria* from the sampled specimens. FLA were isolated more frequently from aerosols and detritus than from biofilms. *Acanthamoeba* has been implicated in a number of diseases including granulomatous amoebic encephalitis [33], a cerebral abscess [34] and chronic keratitis [35]. We also isolated potentially pathogenic *V. avara*, *N. canariensis*, *N. philippinensis* and *A. lenticulata*.

In this study, we detected the *M. ulcerans* IS*2404* target in 4.64% of FLA cultures, significantly more often in *Acanthamoeba* sp. and *Vahlkampfiidae* sp. as has been reported by Gryseels *et al.* [4]. Our inability to detect the targets IS*2606* and KR-B in IS*2404* positive FLA was not surprising since most of the C_T_ values of the IS*2404* target recorded were indicative of low mycobacterial DNA concentrations as observed in other studies carried out in the same sampling sites [4], [36].

The importance of protozoa harboring human-pathogenic bacteria has recently been given much attention, especially in the case of fragile bacteria whose environmental phase would be difficult without the protection of a protozoan host [37]. Moreover, phagocytic protozoans such as FLA strongly resemble vertebrate macrophages; and it has been shown that infection success and internal proliferation is enhanced when bacteria such as *Legionella* and *M. avium* had previously resided inside protozoans [15], [38]. A number of intracellular *Mycobacterium* sp. were isolated from unknown hosts in the specimens, which previously have been shown to live/survive intracellularly in amoebae: *M. simiae*, *M. fortuitum*, *M. septicum*, *M. peregrinum*, *M. terrae*, *M. gordonae*, *M. intracellulare* and *M. lentiflavum* [16], [39]. The most frequently isolated species were: *M. arupense*, *M. fortuitum* and *M. lentiflavum* are potentially pathogenic species. These species were isolated from the environment [40]–[42] and *M. arupense* was isolated from wild African rodents [43]. Intracellular mycobacteria were more frequently isolated from specimens from which we also isolated FLA that may indicate their role as a reservoir for these mycobacteria. Thomas *et al.* [37] also reported a significant association between the presence of amoebae and the presence of mycobacteria. FLA have the additional advantage that they can form cysts, which allow them to persist through harsh periods and be dispersed via the air. It has been suggested that some infections can be acquired by inhaling aerosols containing FLA cells filled with bacteria [44], for example in the case of *Legionella* [45]. The detection of the IS*2404* target in two of the thirteen aerosolized FLA suggests that they may act as vehicles for these mycobacteria. The aerosol transmission hypothesis of *M. ulcerans* was first postulated by Hayman [46], but received little attention, due to the unlikelihood of *M. ulcerans* being airborne as a free-living organism. The possibility that IS*2404* positive mycobacteria including *M. ulcerans* are carried by aerosolized protozoan cysts changes this perspective. More research is, however, needed to explore this transmission route further, and in a subsequent study we investigated the presence of *M. ulcerans* on the skin of healthy inhabitants in the same endemic communities (manuscript in preparation).

BU notification rates varied significantly through time with the highest number of cases recorded in November 2009. BU prevalence has increased during the last quarter of the year in this locality as well as in some endemic regions of Ghana (data not shown); in this case there was no community awareness during this period. BU notification rates correlated positively with detection frequency of mycobacterial DNA in FLA cultures but not with detection of IS*2404* in FLA cultures, suggesting that detection of IS*2404* in FLA cannot predict concurrent BU incidence. However, the time series of this data set was not long enough to test for a potential lagging phase between peaks of IS*2404* detection in FLA and BU incidence.

Similar to our previous study [4], over the time course of experimental infection of *A. castellanii* with *M. ulcerans*, *M. ulcerans* is present within amoebae for up to 28 days albeit at low levels. In addition, both electron microscopy and standard fluorescence microscopy revealed the presence of intracellular bacteria within cysts at 22 days post infection (Figure 4I–J). This is not unexpected due to the previously demonstrated presence and survival of a variety of environmental mycobacteria in cysts [39].

The persistence of strong GFP fluorescence of *M. ulcerans* within *A. castellanii* throughout the experiment indicated that the mycolactone polyketide synthases genes are abundantly expressed intracellularly, as GFP gene expression is under the control of the *mlsA1* promoter [26]. These data also suggest that mycolactone may be produced by the bacteria within the vacuole. While this study was not designed to test the effect of *M. ulcerans* on *A. castellanii*, our observations of fluorescing *M. ulcerans* persisting through 28 days within intact *A. castellanii* suggest that *A. castellanii* is not adversely affected by mycolactone or the presence of the bacteria as was also shown for *A. polyphaga* [4].

The ability of *M. marinum* to persist within amoebae is widely documented [39], [47], [48]. Following the initial time points a decrease in *M. ulcerans*-infected amoebae as reported previously for *M. ulcerans*, *M. shottsii* and *M. pseudoshottsii* [4], [10] was seen which suggests that *M. ulcerans* does not replicate within amoebae and is not as well adapted as *M. marinum* to resist initial amoebic digestion, but is perhaps able to persist once within the vacuolar compartment by preventing lysosomal maturation of the vacuole by as yet undetermined mechanisms.

This study showed the occurrence of the IS*2404* marker in FLA, especially in the genera *Acanthamoeba* and *Vahlkampfiidae*. After co-culturing amoebae and *M. ulcerans* the pathogen persisted at low levels suggesting that it probably only transiently occupies FLA and that it is unlikely that protozoa are a long-term reservoir for this pathogen. While the data we present here confirm that FLA can host mycobacteria that harbor the IS*2404* marker (including *M. ulcerans*), the lack of predictive power of detection of IS*2404* positive FLA in predicting BU notifications suggests FLA are not directly involved in transmission of *M. ulcerans* to humans. We suggest future work should focus on reservoirs that act as *M. ulcerans* amplifiers that link protozoans with humans.

## Supporting Information

Table S1 Real-time PCR C_T_ values of IS*2404* target in FLA cultures.
(DOCX)

Table S2 Identification of FLA per sample and sampling site.
(DOCX)

Table S3 Identification of intracellular mycobacteria per sample and sampling site.
(DOCX)

Video S1 Fluorescing bacteria in *A. castelanii* at 24 h post infection.
(ZIP)
