# Supplementary material for: Investigating the Role of Free-living Amoebae as a Reservoir for Mycobacterium ulcerans
Source: PLoS Negl Trop Dis. 2014 Sep 4;8(9):e3148. doi: 10.1371/journal.pntd.0003148 (PMC4154674; doi:10.1371/journal.pntd.0003148)
Supplement: Table S1 — Real-time PCR CT values of IS2404 target in FLA cultures. (DOCX) [file pntd.0003148.s001.docx]

Table S1. Real-time PCR C_T_ values of IS*2404* target in FLA cultures

| **DNA extract** | **Community** | **Habitat** | **C_T_ IS*2404*** | **C_T_ IS*2606*** | **C_T_KR-B** |
| --- | --- | --- | --- | --- | --- |
| *Acanthamoeba* sp*.* and *Vahlkampfiidae* sp. (99% identical to *Vahlkampfia avara*) | Ananekrom site 2-upper | Biofilm plant | 37.37 | ND | ND |
| *Acanthamoeba lenticulata* | Ananekrom site 3 | Detritus | 30.96 | ND | ND |
| FLA | Serebouso | Biofilm plant | 36.78 | ND | ND |
| *Acanthamoeba* sp*.* and *Vahlkampfiidae avara* | Dukusen | Detritus | 35.73 | ND | ND |
| *Acanthamoeba* sp. | Nshyieso site 2 | Biofilm trunk | 37.14 | ND | ND |
| *Acanthamoeba* sp*.* | Mageda | Detritus | 37.48 | ND | ND |
| *Vahlkampfiidae* sp*.* (92% identical to *V. inornata*) | Pataban | Detritus | 35.94 | ND | ND |
| FLA | Ananekrom site 1 | Biofilm trunk | 30.22 | ND | ND |
| FLA | Serebouso | Biofilm plant | 29.72 | ND | ND |
| *Vahlkampfiidae* and *Naegleria* | Bebuso site 1 | Biofilm plant | 29.46 | ND | ND |
| *Vahlkampfiidae* | Serebouso | Aerosols | 36.28 | ND | ND |
| *Acanthamoebae* and *Vahlkampfiidae* | Serebouso | Aerosols | 37.05 | ND | ND |
| *Vahlkampfiidae* | Dukusen | Biofilm trunk | 35.83 | ND | ND |
| *Acanthamoebae* | Bebuso site 2 | Water | 36.83 | ND | ND |
| *Vahlkampfiidae* | Bebuso site 2 | Biofilm plant | 35.96 | ND | ND |
| *Acanthamoeba lenticulata* and *Naegleria* | Bebuso site 1 | Biofilm trunk | 36.84 | ND | ND |
| *Acanthamoebae* | Bebuso site 1 | Biofilm trunk | 36.27 | ND | ND |
| *Acanthamoebae*, *Vahlkampfiidae* and *Naegleria* | Ananekrom site 1 | Water | 37.49 | ND | ND |
| *Naegleria* sp*.* (93% identical to *N. lovaniensis*) and *Vahlkampfiidae* | Ananekrom site 1 | Biofilm plant | 35.99 | ND | ND |
| *Acanthamoeba sp*., *Vahlkampfiidae* and *Naegleria* | Ananekrom site 1 | Biofilm plant | 38.05 | ND | ND |
| *Acanthamoeba* sp. and *Naegleria* | Nshyieso site 2 | Biofilm plant | 36.77 | ND | ND |
| *Vahlkampfiidae* | Nshyieso site 2 | Biofilm plant | 37.24 | ND | ND |
| *Acanthamoeba* sp. and *Vahlkampfiidae* | Nshyieso site 2 | Biofilm trunk | 37.10 | ND | ND |
| *Acanthamoeba* sp*.* (94% identical to *A. lenticulata*) and *Vahlkampfiidae* | Nshyieso site 2 | Biofilm trunk | 37.07 | ND | ND |
| *Acanthamoebae* and *Vahlkampfiidae* | Nshyieso site 2 | Biofilm trunk | 37.28 | ND | ND |

ND-Not detected
