# Supplementary material for: Investigating the Role of Free-living Amoebae as a Reservoir for Mycobacterium ulcerans
Source: PLoS Negl Trop Dis. 2014 Sep 4;8(9):e3148. doi: 10.1371/journal.pntd.0003148 (PMC4154674; doi:10.1371/journal.pntd.0003148)
Supplement: Table S2 — Identification of FLA per sample and sampling site. (DOCX) [file pntd.0003148.s002.docx]

Table S2. Identification of FLA per sample and sampling site

| Village | Identification of amoebae | Type of sample | | | | | Total |
| --- | --- | --- | --- | --- | --- | --- | --- |
|  |  | Aerosol | Biofilm plant | Biofilm trunk | Detritus | Water |  |
| Ananekrom site 1 | *L. wacamawensis* |  | 0 | 1 | 0 | 0 | 1 |
|  | *T. entericus* |  | 0 | 0 | 1 | 0 | 1 |
|  | *V. avara* |  | 0 | 0 | 1 | 2 | 3 |
|  | *V.* sp. strain AK-2007 |  | 0 | 0 | 2 | 0 | 2 |
|  | *Uncultured vahlk clone 38-2* |  | 0 | 0 | 0 | 1 | 1 |
|  | *Acanthamoeba* strain AM22 |  | 0 | 1 | 0 | 0 | 1 |
|  | Total (%) |  | 0/3 (0) | 2/4 (50.00) | 4/6 (66.67) | 3/4 (75.00) | 9/17 (52.94) |
| Ananekrom site 2-upper | *V. avara* |  | 1 | 2 | 0 |  | 3 |
|  | *Acanthamoeba sp.* |  | 0 | 0 | 1 |  | 1 |
|  | Total (%) |  | 1/1 (100) | 2/1 (200) | ½ (50.00) |  | 4/4 (100) |
| Ananekrom site 2-lower | *N. philippinensis,* strain RNG292 (AB332194.1) |  | 0 | 0 | 1 |  | 1 |
|  | *N. philippinensis* |  | 0 | 0 | 1 |  | 1 |
|  | Total (%) |  | 0/1 (0) | 0/1 (0) | 2/1 (200) |  | 2/3 (66.67) |
| Ananekrom site 3 | *V.* sp. strain AK-2007 |  | 2 | 0 | 0 | 0 | 2 |
|  | *A. lenticulata* |  | 0 | 0 | 2 | 0 | 2 |
|  | Total (%) |  | 2/2 (100) | 0/2 (0) | 2/2 (100) | 0/1 (0) | 4/7 (57.14) |
| Bebuso site 1 | *T. thorntoni, aberdonicus, jugosus* |  | 0 | 0 | 1 | 0 | 1 |
|  | *T. wacamawensis* |  | 0 | 1 | 0 | 0 | 1 |
|  | *V. ciguana* |  | 0 | 1 | 0 | 0 | 1 |
|  | *N. philippinensis* |  | 1 | 0 | 0 | 0 | 1 |
|  | *Acanthamoeba sp.* |  | 0 | 0 | 0 | 1 | 1 |
|  | Total (%) |  | 1/3 (33.33) | 2/3 (66.67) | 1/3 (33.33) | 1/1 (100) | 5/10 (50.00) |
| Dukusen | *N. canariensis* |  | 2 | 0 | 0 | 0 | 2 |
|  | *Uncultured vahlk clone 38-2* |  | 0 | 1 | 0 | 0 | 1 |
|  | *V. avara* |  | 0 | 0 | 4 | 0 | 4 |
|  | *V. inornata* |  | 0 | 2 | 0 | 0 | 2 |
|  | *V.* sp. strain AK-2007 |  | 2 | 0 | 2 | 0 | 4 |
|  | *Uncultured vahlk clone 40-1 EU812490* |  | 0 | 1 | 0 | 0 | 1 |
|  | *Acanthamoeba sp.* |  | 0 | 1 | 0 | 0 | 1 |
|  | *Acanthamoeba* sp. strain PN14 |  | 0 | 0 | 1 | 0 | 1 |
|  | *Acanthamoeba* sp. strain S36 & S22 |  | 0 | 0 | 1 | 0 | 1 |
|  | Total (%) |  | 4/9 (44.44) | 5/7 (71.43) | 8/9 (88.89) | 0/2 (0) | 17/27 (62.96) |
| Mageda | *S. lipophora* |  | 1 | 0 | 0 | 0 | 1 |
|  | *T. entericus* |  | 0 | 1 | 0 | 0 | 1 |
|  | *Uncultured vahlk clone 38-2* |  | 0 | 0 | 1 | 0 | 1 |
|  | *N. canariensis* |  | 0 | 2 | 0 | 0 | 2 |
|  | *Naegleria* sp. strain PNTL |  | 0 | 0 | 1 | 0 | 1 |
|  | *A. lenticulata* |  | 0 | 0 | 1 | 0 | 1 |
|  | Total (%) |  | 1/5 (20.00) | 3/5 (60.00) | 3/5 (60.00) | 0/1 (0) | 7/16 (43.75) |
| Nshyieso site 1 | *T. thorntoni, aberdonicus, jugosus* |  | 2 |  | 3 | 0 | 5 |
|  | *V.* sp. strain AK-2007 |  | 1 |  | 0 | 0 | 1 |
|  | *A. lenticulata* |  | 0 |  | 1 | 0 | 1 |
|  | Total (%) |  | 3/3 (100) |  | 4/3 (133.33) | 0/1 (0) | 7/7 (100) |
| Nshyieso site 2 | *N. clarki, RNG474* |  | 0 | 0 | 2 | 0 | 2 |
|  | *Uncultured vahlk clone 38-2* |  | 2 | 0 | 0 | 0 | 2 |
|  | *Uncultured vahlk clone 38-2 EU812478* |  | 0 | 0 | 1 | 0 | 1 |
|  | *Uncultured vahlk clone 38-2 EU812479* |  | 0 | 0 | 1 | 0 | 1 |
|  | *A. hatchetii* |  | 0 | 2 | 0 | 0 | 2 |
|  | *A. lenticulata* |  | 0 | 0 | 1 | 0 | 1 |
|  | *AcaVNAK05 & AcaVN06*  (GQ905499.1 & GQ397468.1) |  | 0 | 0 | 1 | 0 | 1 |
|  | *Acanthamoeba sp.* |  | 0 | 0 | 1 | 0 | 1 |
|  | Total (%) |  | 2/5 (40.00) | 2/8 (25.00) | 7/6 (116.67) | 0/2 (0) | 11/21 (52.38) |
| Pataban | *V. ciguana* |  | 0 | 1 | 0 | 0 | 1 |
|  | *V. inornata* |  | 0 | 0 | 1 | 0 | 1 |
|  | *N. philippinensis* |  | 1 | 0 | 3 | 0 | 4 |
|  | *N. canariensis* |  | 0 | 1 | 0 | 0 | 1 |
|  | *Naegleria* sp. strain RNG338 |  | 1 | 0 | 0 | 0 | 1 |
|  | *Acanthamoeba sp.* |  | 0 | 1 | 0 | 0 | 1 |
|  | *Acanthamoeba sp. (stevnsoni/hatchetii)* |  | 0 | 0 | 1 | 0 | 1 |
|  | Total (%) |  | 2/4 (50.00) | 3/6 (50.00) | 5/5 (100) | 0/1 (0) | 10/16 (62.50) |
| Serebouso | *Learamoeba* sp. strain RLA498, *T. Entericus* |  | 0 | 0 | 2 | 0 | 2 |
|  | *T. Entericus* |  | 0 | 0 | 2 | 1 | 3 |
|  | *V. sp. SK1* |  | 1 | 1 | 0 | 0 | 2 |
|  | *N. americana* |  | 0 | 1 | 0 | 0 | 1 |
|  | *A. lenticulata* |  | 1 | 1 | 2 | 0 | 4 |
|  | *Acanthamoeba sp.* |  | 0 | 1 | 2 | 0 | 3 |
|  | *Acanthamoeba* strain AM22 |  | 1 | 0 | 0 | 0 | 1 |
|  | Total (%) |  | 3/10 (30.00) | 4/9 (44.44) | 8/7 (114.29) | ½ (50.00) | 16/28 (57.14) |
