# Supplementary material for: Investigating the Role of Free-living Amoebae as a Reservoir for Mycobacterium ulcerans
Source: PLoS Negl Trop Dis. 2014 Sep 4;8(9):e3148. doi: 10.1371/journal.pntd.0003148 (PMC4154674; doi:10.1371/journal.pntd.0003148)
Supplement: Table S3 — Identification of intracellular mycobacteria per sample and sampling site. (DOCX) [file pntd.0003148.s003.docx]

Table S3. Identification of intracellular mycobacteria per sample and sampling site

| Village | Identification of mycobacteria | Type of sample | | | | | Total |
| --- | --- | --- | --- | --- | --- | --- | --- |
|  |  | Aerosol | Biofilm plant | Biofilm trunk | Detritus | Water |  |
| Ananekrom site 1 | *M. arupense* |  | 6 | 3 | 2 | 4 | 15 |
|  | *M. fortuitum* |  | 1 | 0 | 0 | 0 | 1 |
|  | *M. lentiflavum* |  | 0 | 1 | 0 | 0 | 1 |
|  | *M. nonchromogenicum* |  | 0 | 2 | 0 | 0 | 2 |
|  | *M. terrae* |  | 0 | 3 | 0 | 1 | 4 |
|  | Total (%) |  | 7/38 (18.42) | 9/32 (28.13) | 2/4 (50) | 5/11 (45.45) | 23/85 (27.06) |
| Ananekrom site 2-upper | *M. arupense* |  | 0 | 1 | 0 |  | 1 |
|  | *M. peregrinum/septicum* |  | 1 | 0 | 0 |  | 1 |
|  | Total (%) |  | 1/1 (100) | 1/1 (100) | 0/1 (0) |  | 2/3(66.67) |
| Ananekrom site 2-lower | Total (%) |  | 0/1 (0) | 0/1 (0) | 0/1 (0) |  | 0/3 (0) |
| Ananekrom site 3 | *M. arupense* |  | 0 | 1 | 0 | 0 | 1 |
|  | *M. fortuitum* |  | 0 | 1 | 0 | 0 | 1 |
|  | Total (%) |  | 0/2 (0) | 2/2 (100) | 0/2 (0) | 0/1 (0) | 2/7 (28.57) |
| Bebuso site 1 | *M. IWGMT* 90143 | 0 | 0 | 0 | 0 | 1 | 1 |
|  | *M. arupense* | 0 | 2 | 1 | 1 | 1 | 5 |
|  | *M. branderi* | 0 | 1 | 0 | 0 | 0 | 1 |
|  | *M. fortuitum* | 0 | 0 | 1 | 0 | 0 | 1 |
|  | *M. lentiflavum* | 0 | 1 | 0 | 0 | 0 | 1 |
|  | *M. nebraskense* | 0 | 1 | 0 | 0 | 0 | 1 |
|  | *M. nonchromogenicum* | 0 | 0 | 1 | 0 | 0 | 1 |
|  | *M. paraffinicum/scrofulaceum* | 0 | 1 | 0 | 0 | 0 | 1 |
|  | *M. peregrinum* | 0 | 1 | 0 | 0 | 0 | 1 |
|  | *M. simiae/arupense* | 0 | 0 | 1 | 0 | 0 | 1 |
|  | *M. terrae* | 0 | 1 | 0 | 0 | 0 | 1 |
|  | Total (%) | 0/1 (0) | 8/26 (30.76) | 4/28 (14.29) | 1/3 (33.33) | 2/7 (28.57) | 15/65 (23.08) |
| Bebuso site 2 | *M. arupense* | 0 | 0 | 2 |  | 0 | 2 |
|  | *M. celatum* | 0 | 2 | 0 |  | 0 | 2 |
|  | *M. interjectum* | 0 | 1 | 0 |  | 0 | 1 |
|  | *M. interjectum/conspicuum* | 0 | 0 | 1 |  | 0 | 1 |
|  | *M. lentiflavum* | 0 | 1 | 0 |  | 0 | 1 |
|  | *M. paraffinicum* | 0 | 1 | 0 |  | 0 | 1 |
|  | Total (%) | 0/2 (0) | 5/15 (33.33) | 3/9 (33.33) |  | 0/2 (0) | 8/28 (28.57) |
| Dukusen | *M. alvei* | 0 | 1 | 0 | 0 | 0 | 1 |
|  | *M. arupense* | 0 | 2 | 3 | 3 | 0 | 8 |
|  | *M. asiaticum* | 0 | 1 | 0 | 0 | 0 | 1 |
|  | *M. branderi* | 0 | 0 | 1 | 0 | 0 | 1 |
|  | *M. branderi/simiae* | 0 | 0 | 0 | 0 | 1 | 1 |
|  | *M. cookie* | 0 | 0 | 2 | 0 | 0 | 2 |
|  | *M. gilvum* | 0 | 0 | 1 | 0 | 0 | 1 |
|  | *M. gordonae* | 0 | 2 | 0 | 0 | 0 | 2 |
|  | *M. gordonae/fortuitum* | 0 | 1 | 0 | 0 | 0 | 1 |
|  | *M. intracellulare* | 0 | 0 | 1 | 0 | 0 | 1 |
|  | *M. intracellulare/simiae* | 0 | 0 | 1 | 0 | 0 | 1 |
|  | *M. lentiflavum* | 0 | 1 | 0 | 0 | 0 | 1 |
|  | *M. nebraskense* | 0 | 0 | 0 | 0 | 1 | 1 |
|  | *M. paraffinicum* | 0 | 1 | 0 | 0 | 0 | 1 |
|  | *M. scrofulaceum* | 0 | 2 | 0 | 0 | 0 | 2 |
|  | *M. simiae* | 0 | 1 | 0 | 0 | 0 | 1 |
|  | Total (%) | 0/4 (0) | 12/49 (24.49) | 9/39 (23.08) | 3/8 (37.5) | 2/10 (20.0) | 26/110 (23.64) |
| Mageda | *M. IWGMT* 90143 |  | 1 | 0 | 0 | 0 | 1 |
|  | *M. alvei* |  | 0 | 1 | 0 | 0 | 1 |
|  | *M. arupense* |  | 0 | 0 | 2 | 0 | 2 |
|  | *M. peregrinum/septicum* |  | 0 | 0 | 1 | 0 | 1 |
|  | *M. simiae* |  | 1 | 0 | 0 | 0 | 1 |
|  | *Mycobacterium sp.* |  | 1 | 0 | 0 | 0 | 1 |
|  | Total (%) |  | 3/5 (60) | 1/5 (20) | 3/5 (60) | 0/1 (0) | 7/16 (43.75) |
| Nshyieso site 1 | Total (%) |  | 0/3 (0) |  | 0/2 (0) | 0/1 (0) | 0/6 (0) |
| Nshyieso site 2 | *M. arupense* | 0 | 3 | 3 | 0 | 1 | 7 |
|  | *M. fortuitum* | 0 | 1 | 1 | 0 | 1 | 3 |
|  | *M. fortuitum/arupense* | 0 | 1 | 0 | 0 | 0 | 1 |
|  | *M. gordonae* | 0 | 0 | 1 | 0 | 0 | 1 |
|  | *M. peregrinum/septicum* | 0 | 1 | 0 | 0 | 0 | 1 |
|  | *M. scrofulaceum* | 0 | 0 | 0 | 0 | 1 | 1 |
|  | *M. simiae* | 0 | 1 | 0 | 0 | 0 | 1 |
|  | *M. terrae* | 0 | 0 | 0 | 1 | 0 | 1 |
|  | Total (%) | 0/1 (0) | 7/38 (18.42) | 5/39 (12.82) | 1/6 (16.67) | 3/9 (33.33) | 16/93 (17.2) |
| Pataban | *M. arupense* |  | 1 | 1 | 1 | 0 | 3 |
|  | *M. fortuitum* |  | 1 | 0 | 0 | 0 | 1 |
|  | *M. peregrinum/septicum* |  | 0 | 0 | 1 | 0 | 1 |
|  | Total (%) |  | 2/5 (40) | 1/5 (20) | 2/5 (40) | 0/1 (0) | 5/16 (31.25) |
| Serebouso | *M. arupense* | 0 | 5 | 6 | 1 | 0 | 12 |
|  | *M. asiaticum* | 0 | 0 | 0 | 0 | 1 | 1 |
|  | *M. branderi* | 0 | 0 | 1 | 0 | 0 | 1 |
|  | *M. cookie* | 0 | 1 | 0 | 0 | 0 | 1 |
|  | *M. fortuitum* | 0 | 1 | 1 | 1 | 0 | 3 |
|  | *M. intracellulare* | 0 | 2 | 0 | 0 | 0 | 2 |
|  | *M. lentiflavum* | 0 | 0 | 0 | 0 | 2 | 2 |
|  | *M. scrofulaceum* | 0 | 1 | 0 | 0 | 0 | 1 |
|  | *M. terrae* | 0 | 1 | 3 | 0 | 0 | 4 |
|  | Total (%) | 0/4 (0) | 11/40 (27.5) | 11/44 (25) | 2/8 (25) | 3/10 (30) | 27/106 (25.47) |
